# Automatic glaucoma detection based on transfer induced attention network

**DOI:** 10.1186/s12938-021-00877-5

**Published:** 2021-04-23

**Authors:** Xi Xu, Yu Guan, Jianqiang Li, Zerui Ma, Li Zhang, Li Li

**Affiliations:** 1 Faculty of Information Technology, Beijing University of Technology, Beijing, China; 2 Beijing Tongren Hospital, Capital Medical University, Beijing, China; 3 Beijing Children’s Hospital, Capital Medical University, Beijing, China

**Keywords:** Automatic glaucoma diagnosis, Transfer learning, Deep learning, Attention mechanism

## Abstract

**Background:**

Glaucoma is one of the causes that leads to irreversible vision loss. Automatic glaucoma detection based on fundus images has been widely studied in recent years. However, existing methods mainly depend on a considerable amount of labeled data to train the model, which is a serious constraint for real-world glaucoma detection.

**Methods:**

In this paper, we introduce a transfer learning technique that leverages the fundus feature learned from similar ophthalmic data to facilitate diagnosing glaucoma. Specifically, a Transfer Induced Attention Network (TIA-Net) for automatic glaucoma detection is proposed, which extracts the discriminative features that fully characterize the glaucoma-related deep patterns under limited supervision. By integrating the channel-wise attention and maximum mean discrepancy, our proposed method can achieve a smooth transition between general and specific features, thus enhancing the feature transferability.

**Results:**

To delimit the boundary between general and specific features precisely, we first investigate how many layers should be transferred during training with the source dataset network. Next, we compare our proposed model to previously mentioned methods and analyze their performance. Finally, with the advantages of the model design, we provide a transparent and interpretable transferring visualization by highlighting the key specific features in each fundus image. We evaluate the effectiveness of TIA-Net on two real clinical datasets and achieve an accuracy of 85.7%/76.6%, sensitivity of 84.9%/75.3%, specificity of 86.9%/77.2%, and AUC of 0.929 and 0.835, far better than other state-of-the-art methods.

**Conclusion:**

Different from previous studies applied classic CNN models to transfer features from the non-medical dataset, we leverage knowledge from the similar ophthalmic dataset and propose an attention-based deep transfer learning model for the glaucoma diagnosis task. Extensive experiments on two real clinical datasets show that our TIA-Net outperforms other state-of-the-art methods, and meanwhile, it has certain medical value and significance for the early diagnosis of other medical tasks.

## Background

Glaucoma is a kind of chronic disease that damages optic nerve of the eye. Due to the difficulty of examination and treatment, patients with glaucoma often suffer from visual impairment or even irreversible blindness. According to research [1], there are 44.7 million people diagnosed with glaucoma worldwide in 2010, and this figure is predicted to increase by about 50% within a decade. The blindness incidence of this disease is nearly one-third, second only to cataract [1, 2]. In China, because of the low medical and domestic economic level, the rate of glaucoma treatment is less than one-tenth [3]. Therefore, early screening is essential to prevent further deterioration in glaucoma patients.

In the medical field, fundus photography is a popular method implemented for early screening of glaucoma. Ophthalmologists clinically detect glaucoma according to certain symptoms, including high intraocular pressure, optic nerve damage, large cup-to-disc ratio, and vision loss [4, 5], which are widely used as diagnostic criteria. However, manual glaucoma assessment is expensive and time-consuming for patients as the professional knowledge of ophthalmology is needed for the whole process. Consequently, there are many studies involved in how to automatically identify glaucoma with computer vision algorithms. The mainstream of these studies are divided into two categories: heuristic methods and deep learning methods.

Heuristic methods mainly utilized the domain expertise to extract features manually [6], including energy-based features [7], local configuration pattern features [8], higher order spectra features [9], and cup-to-disc ratio features [10], etc. However, predefined features need to be extracted artificially, which is a laborious heuristic (requiring professional knowledge) meanwhile largely dependent on experience and luck. Furthermore, these features may oversimplify the problem and be ad hoc, for even experts may omit some important hidden patterns. Therefore, deep learning, which is able to automatically extract hidden features from complicated input images, has developed rapidly in the medical field in recent years [11–14]. Deep learning methods have achieved better performance than heuristic methods, and show feasibility of automatic glaucoma diagnosis. However, these methods extract features based on large labeled data, which is a serious constraint in the medical field. Transfer learning aims to generalize deep learning methods to limited supervision scenario by sharing transferable features learned across multiple datasets [15]. And this technology has been explored and showed superiority in various medical tasks [6, 16, 17]. However, these studies ignore the dataset bias and feature gap, which disturbs the model generalization ability.

In fact, an intuitive idea of transfer process is to find similar general features from efficient source data, and then gradually learn the task-specific features. Thus, this paper proposes an automatic glaucoma detection method from the following aspects:

### General feature

The previous studies mainly rely on non-medical datasets to extract general features for medical tasks [6, 18]. However, different image types across domains enlarge dataset bias, thus reducing the transferability of general features. Therefore, we should ensure image consistency, so that general features are safely transferable to the specific task.

### Specific feature

When deep features transition from general to specific along the network, redundant regions in the fundus image (such as the edge regions of the eyeball or other glaucoma-unrelated pathological areas) may mislead specific features to focus on the useless information [19]. In this work, what we consider is how to enhance the ability of specific features to extract key pathology areas, which is expected to achieve superior transfer performance.

**Fig. 1:**
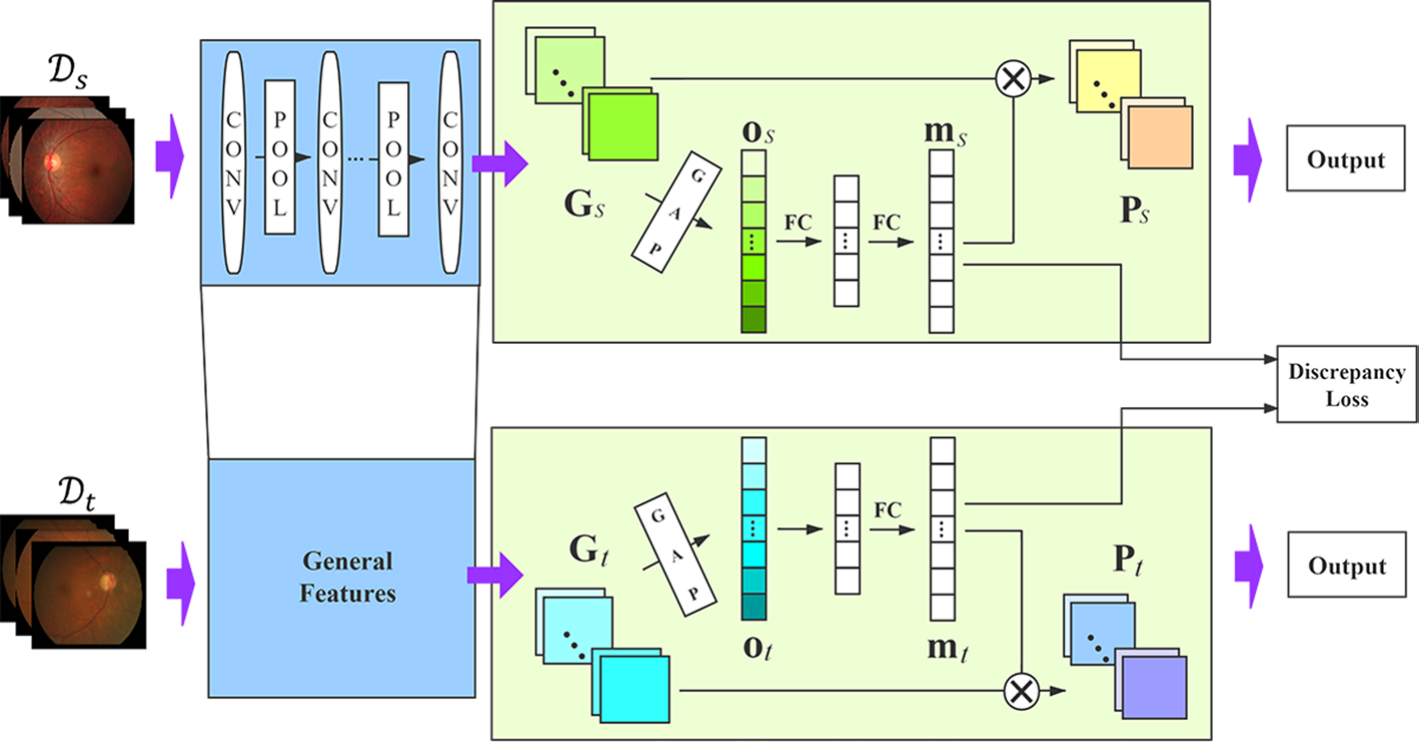
Architecture of our TIA-Net for glaucoma detection

For addressing the above problems, we present a transfer induced attention network (TIA-Net) to reduce the dataset bias and enhance the feature transferability, as shown in Fig. 1. Specifically, we first select similar ophthalmic fundus images rather than from non-medical data to extract general features, thus reducing data differences. Then, the channel-wise attention and maximum mean discrepancy are adopted to make specific features focused on the key pathological areas rather than other redundant information. By this way, the feature gap between general and specific can be bridged by our proposed method. Finally, we conducted extensive experiments on two real clinical datasets and the results show that our proposed method outperforms other state-of-the-art methods. In general, the contributions of this work can be summarized into two points: (1) We propose a transfer induced attention deep learning network (TIA-Net) for automatic glaucoma diagnosis. A similar ophthalmic dataset is selected as source dataset, such that the transferability of general features can be improved. Meanwhile, the channel-wise attention and maximum mean discrepancy are applied to TIA-Net, which are exploited to refine the general-to-specific feature representations. (2) For evaluation of glaucoma detection, we conducted extensive experiments on two real clinical datasets, and the results prove that the proposed method can effectively capture the discriminative features that better characterize the glaucoma-related hidden patterns under limited supervision.

The rest of the paper is organized as follows. A brief review of the state-of-the-art methods is given on automatic glaucoma detection in the rest of this section. Then, we show and analyze the experimental results in the section “Results” and “Discussion”. In the section “Conclusion”, we conclude our work and present some future topics. Finally, the section Methods introduces the data used and our proposed model.

## Related works

### Heuristic method

**Fig. 2:**
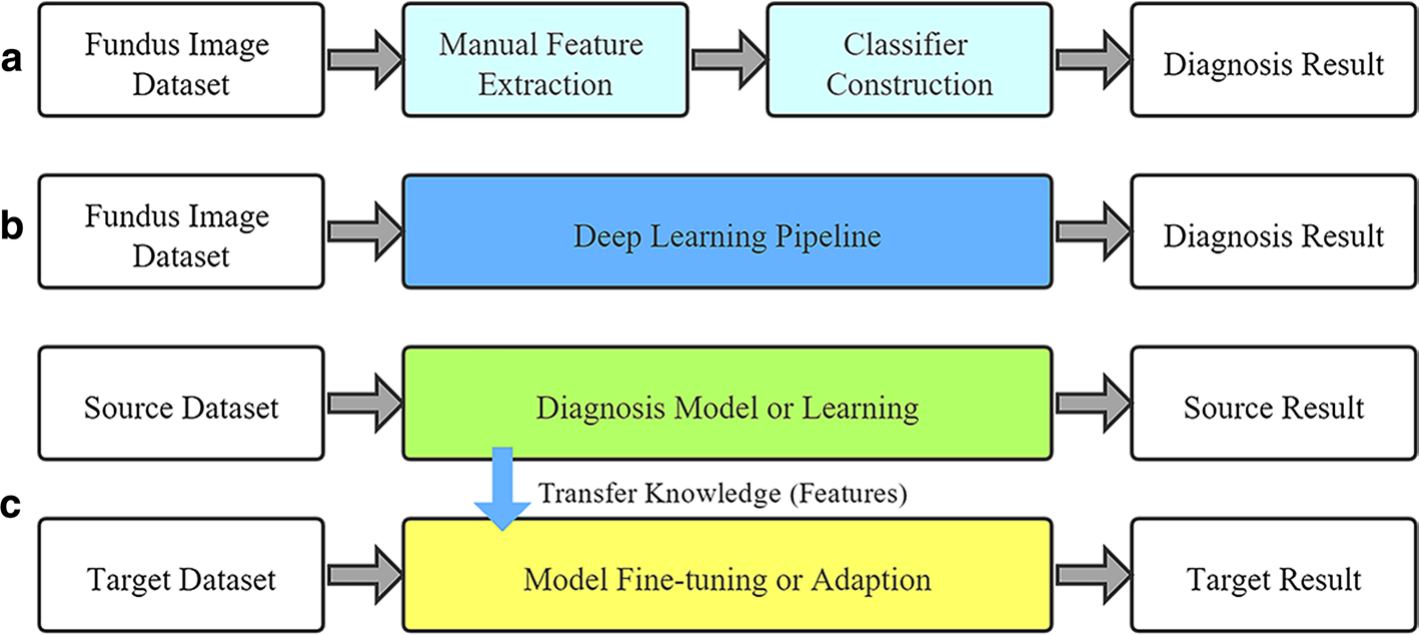
Different automatic glaucoma detection frameworks. **a** Heuristic methods, **b** deep learning methods, and **c** transfer learning methods

Studies on automatic glaucoma detection based on retinal fundus images can basically be divided into two categories: heuristic methods and deep learning methods. Early studies usually use heuristic methods to complete this task that mainly utilized the professional expertise to extract predefined features (shown in Fig. 2a). Nayak J et al. [20] used geometric characteristics (e.g., cup-to-disc ratio, ratio of the distance between optic disc center, and so on) and artificial neural network classifier to predict glaucoma. In [21], Yadav et al. selected texture features of the area around optic cup to improve the detection model performance. In [22], the independent HOS-based features that appended to texture features were served to build SVM model; and the results show its superiority. The work in [23] applied wavelet transformation of fundus images as features and the discriminant analysis promoted with three main algorithms (including support vector machine, random forest, and naïve Bayes) as the classifiers. Besides, there are many studies that designed various other features such as energy-based features [7], local configuration pattern features [8], fast Fourier transform features [24], entropy-based features [25], and gabor transformation features [26].

Although many of heuristic methods show the effectiveness of automatic glaucoma diagnosis, predefined feature sets require a considerate amount of engineering skill and domain expertise, which is time-consuming and laborious. Besides, these manually designing features might be affected by personal subjective factors, for even doctor, experts may omit some important hidden patterns.

### Deep learning method

Deep learning methods, which are able to automatically learn complicated hidden patterns from high-dimensional data (shown in Fig. 2b), have achieved superiority in many studies [27, 28]. Hence, another category of glaucoma detection methods is based on deep learning [11, 13, 14, 19, 29, 30]. Some studies developed deep learning models based on the automatic segmentation of glaucoma-related areas [13, 14]. Zilly et al. [13] and Shankaranarayana et al. [14] proposed to segment optic cup and disc from retinal images using entropy sampling and ensemble learning, and fully convolutional and adversarial networks, respectively. Although these studies extracted some medical features (e.g., cup-to-disc ratio) related to glaucoma, they ignored other useful hidden features on the fundus images. On the other hand, some other studies obtained sufficient rules of glaucoma discrimination directly through deep learning methods [11, 29, 30]. Chen et al. [11] first preprocessed original fundus images and then trained a CNN structure for glaucoma detection. To get better results, Shibata et al. [29] further proposed a deeper CNN model based on ResNet. A multi-stream CNN that combined the global image and the local disc area has been proposed in [30].

However, due to the limited training data, their works are difficult to have high sensitivity and specificity. Recently, Li et al. [19] established a large database of glaucoma-labeled fundus images and developed an attention-based CNN model, improving the performance in glaucoma detection. However, in real applications, especially for those medical tasks, it is difficult or even impossible to collect sufficient manually labeled samples.

### Transfer learning method

Recently, transfer learning mechanism has been successfully applied in deep-learning-based computer vision tasks [16, 17, 31, 32]. Different from traditional machine learning procedure, the motivation of transfer learning is to improve the model performance under the limited target dataset samples by leveraging the knowledge (features) from source dataset (shown in Fig. 2c) [15]. Since deep learning networks are able to learn transferable features across multiple datasets [33], it is helpful to transfer knowledge to exploit the full potential of advances in deep learning on available limited datasets, especially in the medical field. However, there are only a few works considering the application of transfer learning in the CNN model for using fundus images to detect ophthalmic diseases. Orlando et al. [6] shared the CNN weights learned from the ImageNet dataset to train the glaucoma detection model. Christopher et al. [18] further combined transfer learning with several deep learning models to prove its applicability of clinical diagnosis.

These studies have preliminarily explored the effectiveness of transfer learning in the field of automatic glaucoma diagnosis, but they mainly have two limitations: (1) Relying on less-transferable general features which are extracted from the non-medical dataset (e.g., ImageNet dataset). (2) Ignoring the feature gap between general and specific. Therefore, it is reasonable to develop a new transfer learning architecture for fundus image recognition. In this paper, we select similar ophthalmic fundus images to extract general features, and apply the channel-wise attention and maximum mean discrepancy to make a smooth transition of general-to-specific features. Both are jointly employed to enhance the transferability of general and specific features, which is expected to enhance the performance of glaucoma detection.

## Results

### Set of experiments

In our experiment, a tenfold cross-validation is used to evaluate all the methods. During processing, we remove patients’ personal medical information and meanwhile retain the original information as much as possible, since privacy protection for patients is the focus of public attention [34]. After that, we employ data augmentation to reduce overfitting on image data using label-preserving transformations. We then resize all fundus images uniformly to 256 $*$ 256 pixels, since the experimental images have different sizes. To test the generalization ability, we further validate the performance of our proposed method on ORIGA dataset [35].

For the parameter setting in training, we employ step learning policy and initially set the learning rate to $10^{-2}$ for all layers. All models are trained for 100 epochs from scratch, using the weight initialization strategy described in [36]. The units of the output FC layer are changed according to the number of training data’s classes. We set the batch size to 16 and momentum to 0.9. L2 weight decay is applied with penalty multiplier set to $5 * 10^{-4}$ and dropout ratio set to 0.5, respectively. All the experiments are conducted on a workstation with Windows 10, a 3.50 GHz Intel(R) Xeon(R) E5-1620 CPU, and a Nvidia GTX 2080Ti GPU.

For the glaucoma detection task, we adopt four commonly used evaluation criteria to evaluate the performance of classification models, including accuracy, sensitivity, specificity, and area under the curve (AUC). Specifically, the metrics of sensitivity and specificity are defined as follows:

1 $$\begin{aligned}&\text { Sensitivity }=\frac{\text {TP}}{\text {TP}+\text {FN}} \end{aligned}$$

2 $$\begin{aligned}&\text { Specificity }=\frac{\text {TN}}{\text {TN}+\text {FP}}, \end{aligned}$$

where TP, TN, FP, and FN are the numbers of the true-positive glaucoma, true-negative glaucoma, false-positive glaucoma, and false-negative glaucoma, respectively.

### Experimental results

**Fig. 3:**
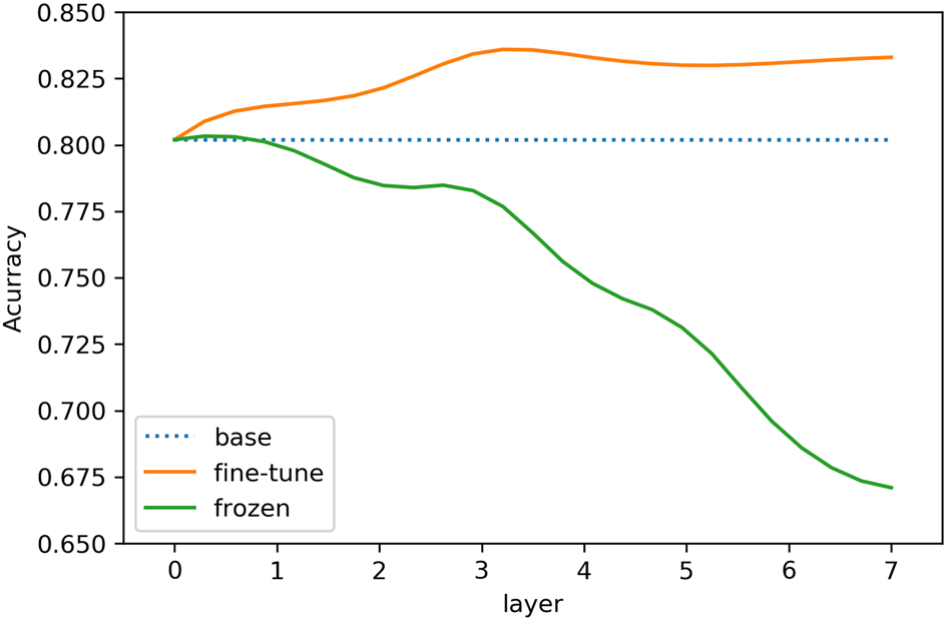
Influence of different transferred layers on accuracy

**Fig. 4:**
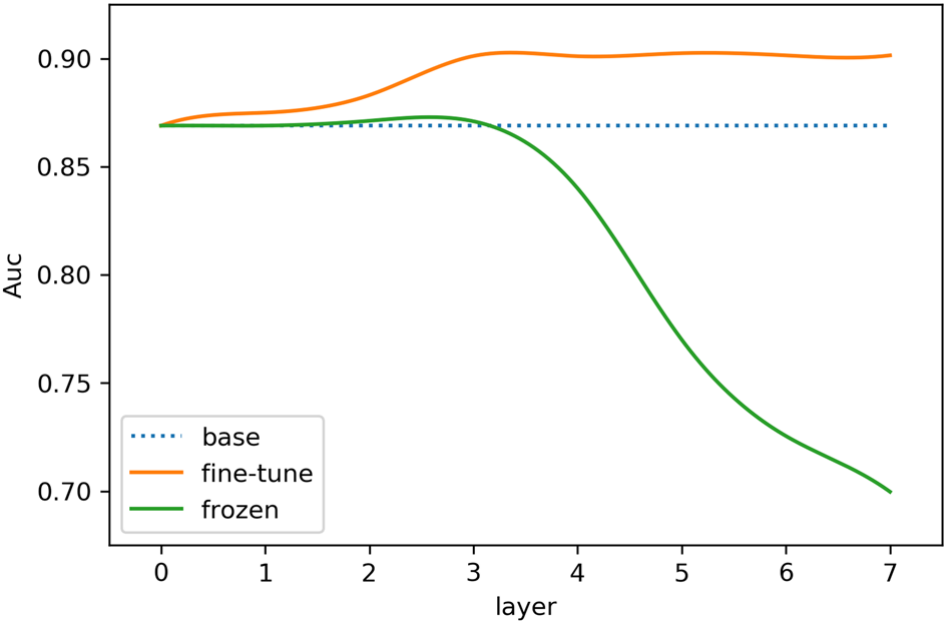
Influence of different transferred layers on AUC

First, we explore the best settings of transferred layers on glaucoma detection. To do a better comparative experiment, we set up two groups of comparative experiments: in one group, the transferred layers have to be frozen consecutively, while the remaining layers are trained along with their weights updated; another group implements fine-tune strategy, means the whole network is updated after transferring. Analyzing the model performance with the frozen parameters of different layers will identify the best transfer network for the target task, i.e., which levels of general to specific features are useful. Figures 3, 4, respectively, show the effects of transferring different layers (our base CNN network has seven layers in total) on the accuracy and Area Under Curve (AUC) of glaucoma classification.

**Fig. 5:**
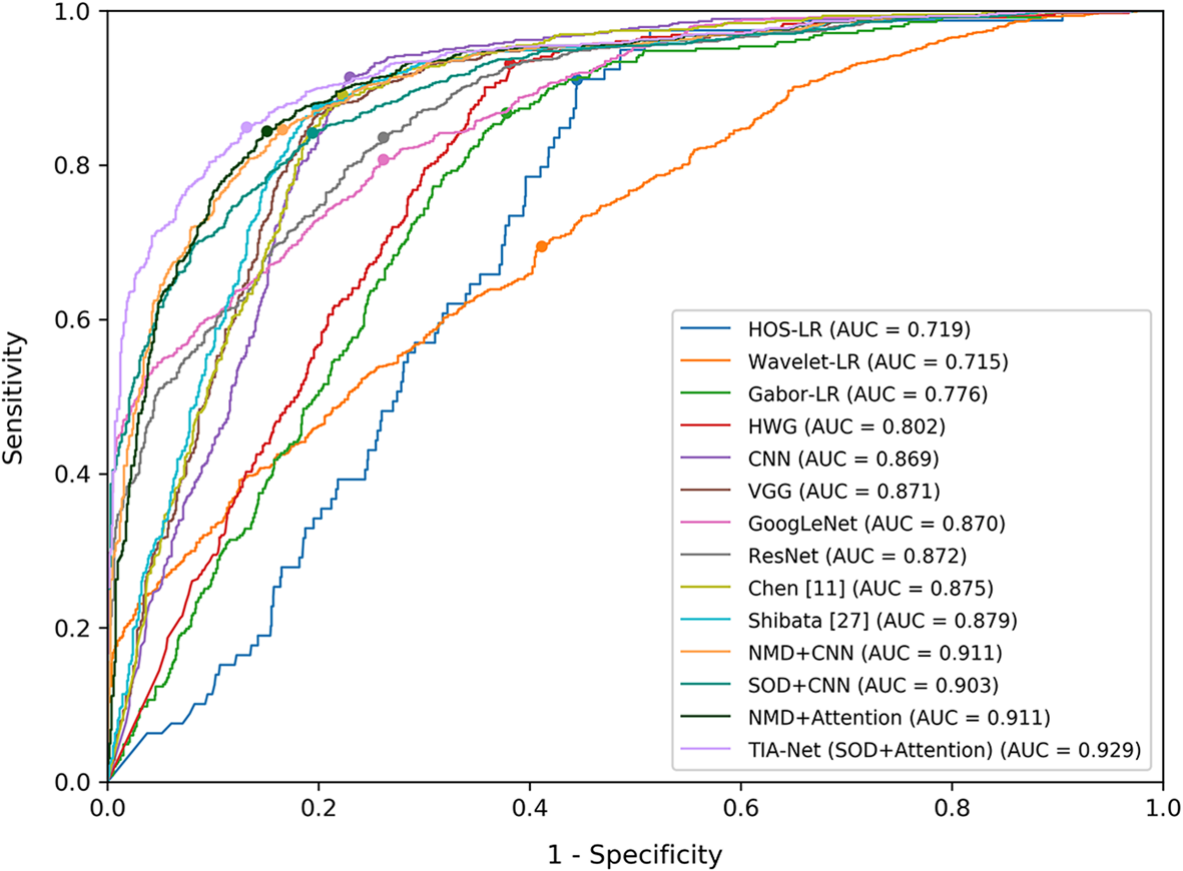
Comparison of ROC curves among different methods (Testing on our database)

**Fig. 6:**
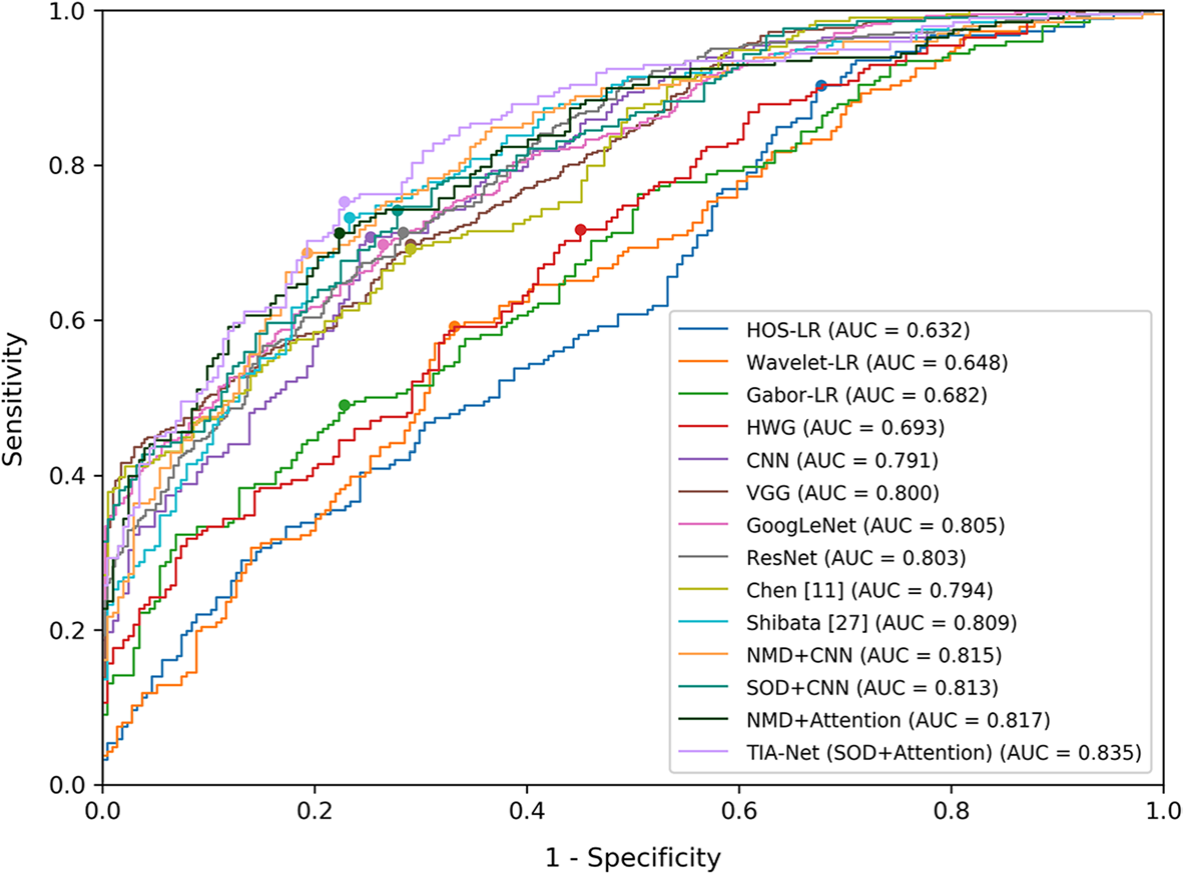
Comparison of ROC curves among different methods (testing on ORIGA database)

**Fig. 7:**
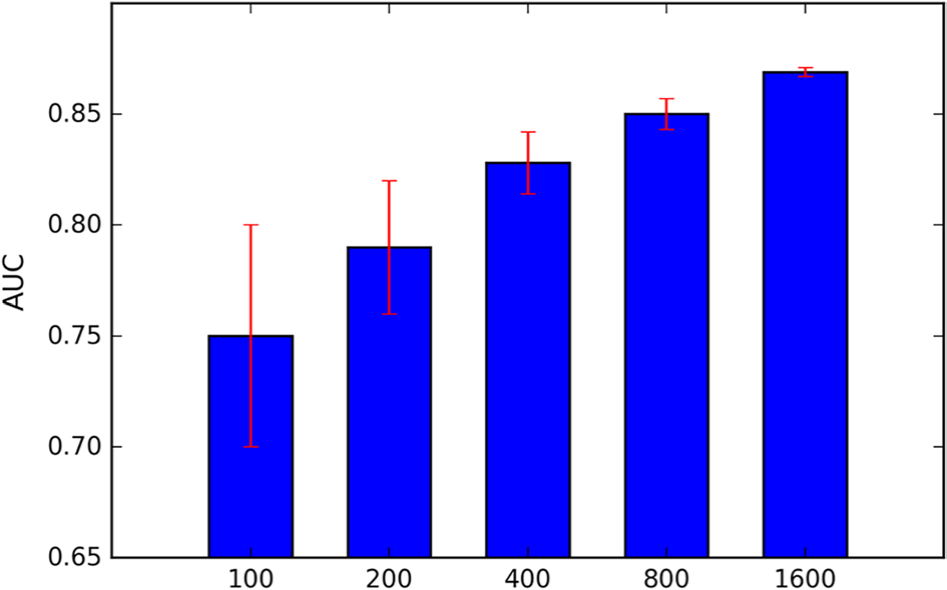
AUC of base CNN model in different numbers of training samples

Second, to fully evaluate the performance of our TIA-Net, we compare it with three benchmark sets: (1) For heuristic methods, we train logistic regression models based on higher order spectra (HOS) [22], discrete wavelet transform [23], Gabor transformation features [26], and the combination of these three handcrafted features, respectively. We denote them as HOS-LR, Wavelet-LR, Gabor-LR, and HWG correspondingly. (2) For classical deep learning methods, we select our base CNN model (CNN) and five other representative models: VGG [37], GoogLeNet [38], ResNet [39], Chen et al. [11], and Shibata et al. [29]. (3) To assess the impact of two main components in TIA-Net on the performance of glaucoma detection, we set up four transfer learning comparisons according to different transfer training procedures and different network structures: NMD + CNN, NMD + Attention, SOD + CNN, and SOD + Attention. For the transfer training procedure, one selects a non-medical dataset (NMD) as the source dataset, i.e., ImageNet dataset, which has been used in [6, 18]; and another uses similar ophthalmic dataset (SOD), i.e., cataract dataset in this paper. For the network structure, one is the base CNN network (CNN), and another is our attention-based network (Attention). Table 1, and Figs. 5 and 6 show the performance results among various glaucoma detection methods in three benchmark sets. Besides, to illustrate the bottleneck caused by insufficient glaucoma training data, the performance of base CNN in different numbers of training sample is shown in Table 2 and Fig. 7.

**Table 1:** Comparison between TIA-Net and models in three benchmark sets

Database	Methods	Acc (%)	Se (%)	Sp (%)	AUC
Ours	HOS-LR	69.9	91.1	55.6	0.719
	Wavelet-LR	68.9	69.5	58.9	0.715
	Gabor-LR	70.5	86.7	62.2	0.776
	HWG	72.1	93.2	61.9	0.802
	CNN	80.2	91.4	77.0	0.869
	VGG	80.7	87.7	79.1	0.871
	GoogLeNet	79.8	80.7	73.8	0.870
	ResNet	81.2	83.6	73.9	0.872
	Chen [11]	80.9	89.1	77.8	0.875
	Shibata [29]	81.7	87.5	80.2	0.879
	NMD+CNN	84.1	84.7	83.4	0.911
	SOD+CNN	83.7	84.2	80.6	0.903
	NMD+Attention	84.5	84.4	84.9	0.911
	TIA-Net (SOD+Attention)	85.7	84.9	86.9	0.929
ORIGA	HOS-LR	63.5	90.3	32.2	0.632
	Wavelet-LR	65.9	59.1	66.8	0.648
	Gabor-LR	67.2	49.0	77.2	0.682
	HWG	68.8	71.7	55.0	0.693
	CNN	70.4	70.7	74.8	0.791
	VGG	70.1	69.8	71.0	0.800
	GoogLeNet	71.8	69.8	73.5	0.805
	ResNet	71.5	71.3	71.7	0.803
	Chen [11]	70.8	69.2	71.0	0.794
	Shibata [29]	73.3	73.2	76.7	0.809
	NMD+CNN	74.5	68.7	80.7	0.815
	SOD+CNN	73.9	80.9	72.2	0.813
	NMD+Attention	74.9	71.2	77.7	0.817
	TIA-Net (SOD+Attention)	76.6	75.3	77.2	0.835

Acc, Se, and Sp represent accuracy, sensitivity, and specificity, respectively

**Table 2:** Performances and variance values of base CNN model in different numbers of training samples

Number of data	100	200	400	800	1600
Accuracy (%)	72.6	75.9	77.2	79.5	80.1
Variance $(10^{-5})$	578.34	113.23	43.21	26.67	15.32
Sensitivity (%)	80.2	83.9	87.9	89.7	91.2
Variance $(10^{-5})$	478.43	101.30	53.98	24.67	19.92
Specificity (%)	70.2	72.5	74.3	75.9	76.8
Variance $(10^{-5})$	438.55	112.23	48.34	25.61	18.45

**Fig. 8:**
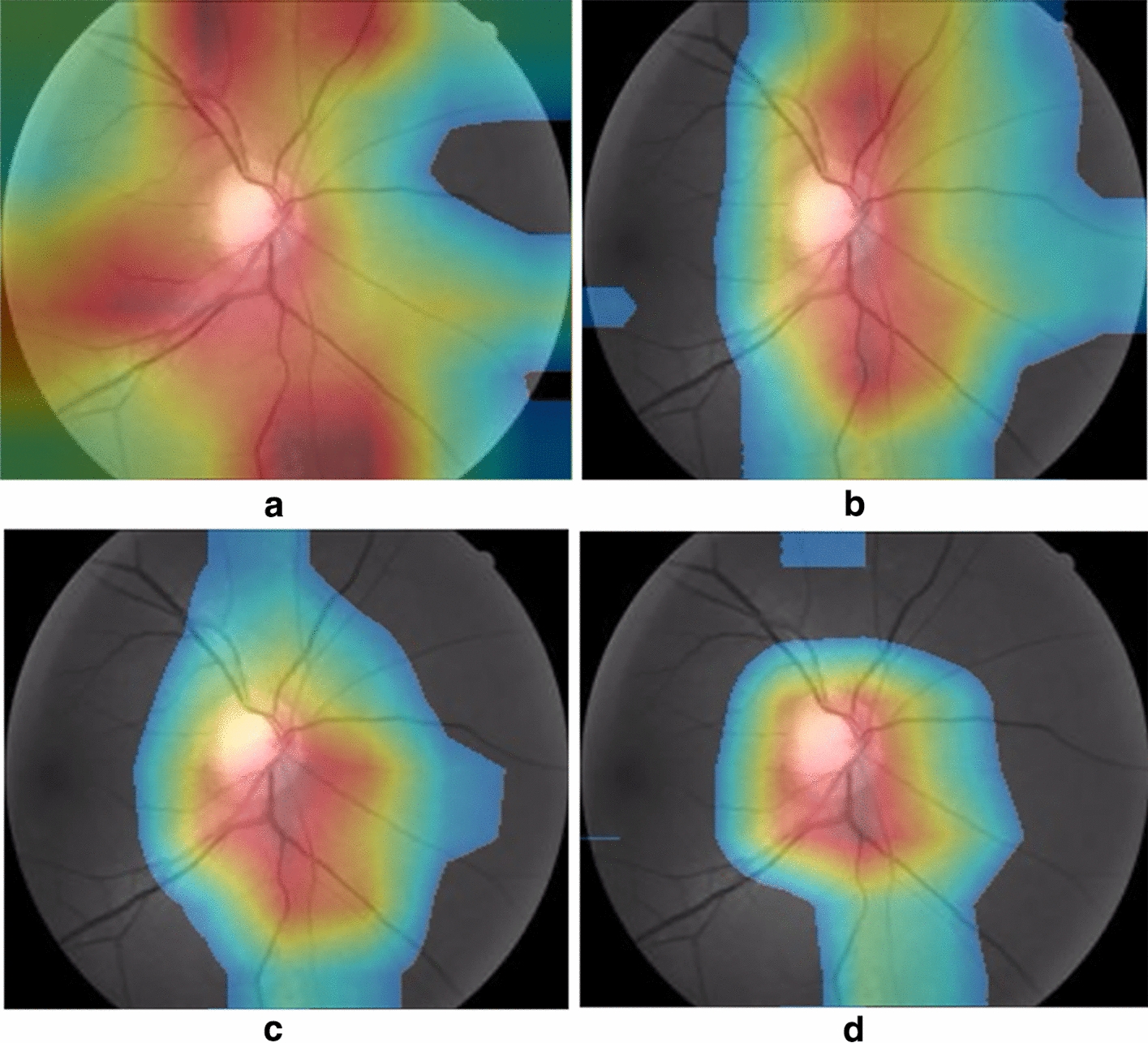
Changes of pathological areas during feature transfer

To prove the effectiveness of TIA-Net for specific feature extraction, we provide a transparent and interpretable process of feature transfer in this part. Specifically, at the end of pre-training and starting to use source and target dataset for training, we visualize the localization changes of pathological area under different training iterations. When the original transferred feature map ${\mathbf {G}}$ is reweighted with the channel attention map ${\mathbf {m}}$, we can get learned specific attention feature ${\mathbf {P}}$ (in Eq. (4)). The specific attention feature ${\mathbf {P}}$ is used to generate these heat maps by masking the input fundus image in different iterations, where warm-colored area indicates high weight region for the detection of glaucoma (e.g., the red area represents most critical region in making the classification, whereas the yellow area is more important than blue). Here, we take a positive sample of glaucoma as a description. As shown in Fig. 8a–d demonstrate the visualized heat maps of transfer processing with the training rounds increased (0, 5th, 15th, and 100th iteration, respectively).

## Discussion

### Effects of different transferred layers on glaucoma detection

According to Figs. 3, 4, we can find the following two points. (1) Transfer learning using fine-tune strategy effectively improves the model performance, and both accuracy and AUC are above the original CNN baseline (the blue curves in Figs. 3, 4). This suggests that the general features from source cataract dataset, including learned gabor features and color blobs, are beneficial to the target glaucoma task. (2) The green curves in both of Figs. 3, 4 (representing the frozen strategy) basically are declined especially from the fourth layer. This could be because the fourth layer is the dividing line between general features and specific features of base CNN model. Since there is a considerable difference between pathological features of the cataract and glaucoma in this experiment, the direct use of the high-layer specific features of the source domain network may cause negative transfer to the target task. For example, large and small blood vessels are sensitive information for cataract classification, but not significantly helpful for glaucoma detection. In summary, although the discriminant features of the two ophthalmic diseases are different, the shallow general features of cataract dataset can be used to supplement the target glaucoma classification task due to the consistent basic features of fundus images. It is proved that this mechanism is helpful to improve the performance at the limited training supervision.

### Evaluation on glaucoma detection

From Table 1, and Figs. 5, 6, we can summarize the following finding: (1) The heuristic methods in benchmark set 1 do not achieve good performance on the two datasets, with accuracies and AUC all around 0.70 (on our database)/0.65 (on the ORIGA database). The reason may be that predefined features in these models are not the best patterns of glaucoma and non-glaucoma cases. (2) It is clearly seen that deep learning methods outperform heuristic methods, which demonstrates that deep learning methods are able to extract better features than heuristic methods. However, we further find that these deep learning methods do not differ greatly in performance. For example, all metrics of these deep learning methods do not exceed 0.81 on the ORIGA database. Hence, we infer that they still have shortcomings in improving the performance, a bottleneck caused by insufficient glaucoma training data. The validity of this hypothesis can be proved by Table 2 and Fig. 7, as the number of training samples has a significant impact on the performance of the deep learning method: the greater the amount of data, the better and more stable the model performs. (3) Although the performance of benchmark set 3 is better than deep learning models in some metrics, the improvement is not significant. This may be because 1) non-medical dataset leads to a poor transferability of general features; 2) irrelevant redundancy influences the extraction of specific features. When both similar ophthalmic dataset and transfer induced attention are introduced, TIA-Net obtains the best performance on our database (85.7% accuracy/0.929 AUC) and the ORIGA database (76.6% accuracy/0.835 AUC), which has about 2% improvement than the best combination in benchmark set 3 (NMD+Attention). It indicates the necessity of the introduction of both similar ophthalmic dataset and transfer induced attention structure, since more latent discriminative information for glaucoma detection can be obtained under limited supervision.

### Pathological area visualization in feature transfer

In the early stage of training, We find that TIA-Net focuses on the optic disc and the blood vessels, which are all pathological areas for cataract screening (shown in Fig. 8a). However, the large and small blood vessels are redundant information for glaucoma detection. As seen in Fig. 8b, c, the pathological areas of specific features have changed significantly with the increasing rounds of training. In particular, the salient areas in the heat maps are gradually concentrated, while the redundant areas are reduced. At the end of training convergence, we find that our TIA-Net accurately locates the optic cup and disc, especially for the pathological areas of the inferior and superior optic disc, which are commonly used by ophthalmologists to diagnose glaucoma (as shown in Fig. 8d). The visualization results indicate that: (1) The appropriate extraction of general features guarantees the transformation of high-level specific features between source and target datasets. (2) Transfer induced attention makes specific features effectively focus on the key pathological areas with reduced redundancy. Both of them jointly ensure the stability of high classification performance. These specific attention features bridge among the diagnosis model and users (including ophthalmologists and patients), leading to a better understanding of our transfer mechanism.

### Future works

In this paper, the non-glaucomatous cases in our dataset cover various ophthalmic diseases, since all fundus images are collected from real-world screening. However, symptoms of other diseases may interfere with feature extraction for glaucoma detection. For example, PPA is a common symptom between high myopia and glaucoma; all important fundus features are fuzzy and visible in severe cataract. This limitation makes the learning task difficult, thus affecting the performance of the model. To address this problem, we will establish a large-scale database that contains more heterogeneous samples and design an auxiliary module to distinguish complicated cases, thus improving the generalization of our method. Besides, we will investigate the transfer patterns of different CNN structures, e.g., residual block and inception architecture, to select the appropriate base network for feature extraction.

## Conclusion

In this paper, we leverage knowledge from similar ophthalmic dataset and propose an attention-based deep transfer learning model for the glaucoma diagnosis task, which includes two main operations: transferring general features from similar ophthalmic dataset and extracting specific features based on transfer induced attention. It is an appropriate combination for automatic glaucoma detection due to two reasons: (1) Since the basic features in fundus images are consistent between source and target datasets, the transferability of general feature would be improved. (2) Although there still exists irrelevant redundancy in the transfer process, the channel-wise attention and the maximum mean discrepancy can adaptively recalibrate the feature mapping of transmission to focus on key glaucoma-related areas. Experiments conducted on two real clinical datasets prove that TIA-Net is particularly efficient and useful in modeling glaucoma detection. In the future work, we plan to conduct comprehensive experiments to investigate the transfer patterns in the different eye diseases and CNN networks.

## Methods

In this section, we introduce the proposed TIA-Net. The framework of TIA-Net is displayed in Fig. 1, and its processes, including two main operations: (1) transferring general features from similar ophthalmic dataset and (2) extracting specific features based on transfer induced attention, are further highlighted as blue and green blocks, respectively, in the figure. To learn general features, we first pre-train base CNN network on labeled cataract dataset and explore the best settings of transferred layers on glaucoma detection. We then transfer general features into TIA-Net to help learn specific features, and optimize the weights according to the loss of Eq. (5) using both source and target data. Note that we rely on mini-batches for training, since large batch sizes will increase the computational cost.

### Data

In the medical field, the digital fundus screening is a popular diagnostic examination, since it is safe and efficient to analyze the changes of hypertensive retinopathy and arteriosclerosis in patients with various eye diseases. The retinal fundus images used in this paper contain two categories: the glaucoma images for target dataset and the cataract images for source dataset, respectively, which are all manually labeled by professional ophthalmologists from Beijing Tongren Eye Center. Subjects in the dataset are mainly from northern China. Among them, the proportion of males is around 48%; the remaining 52% are females. The age range of the subjects in the dataset is from 10 to 90.

**Fig. 9:**
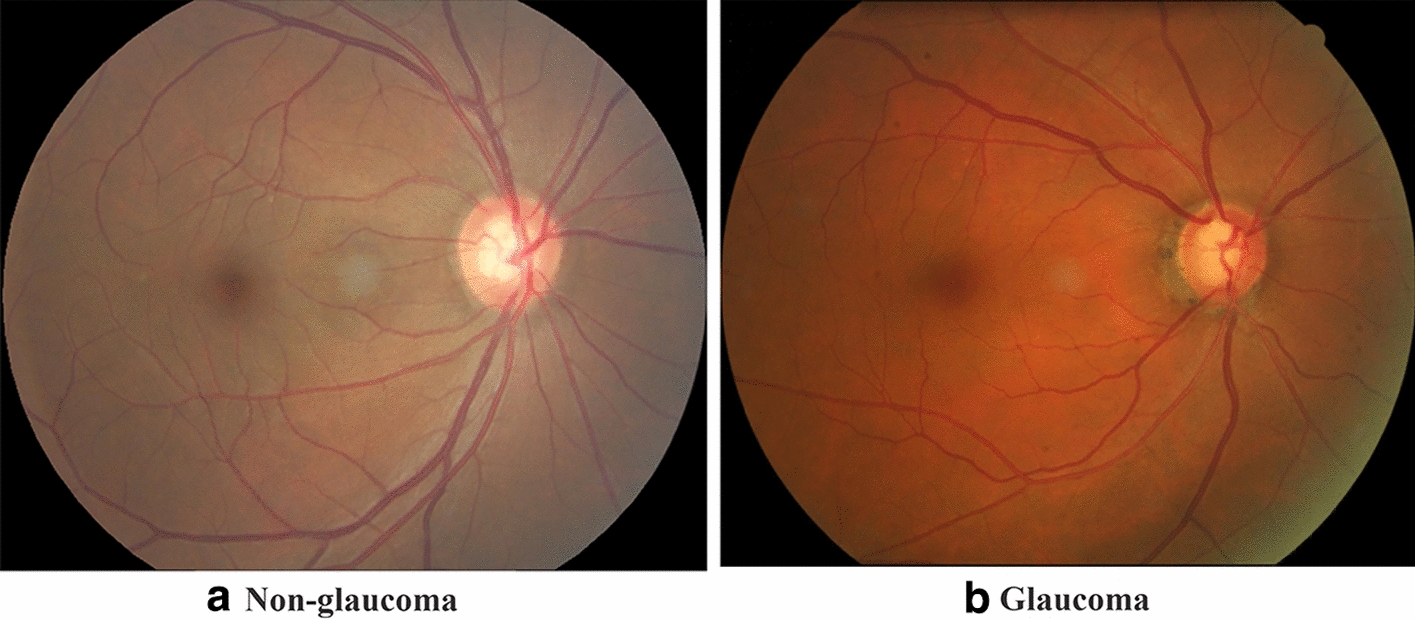
Fundus images of non-glaucoma and glaucoma cases

The glaucoma dataset contains 1882 retinal fundus images, including non-glaucoma (1005) and glaucoma (877), where the uniform size of each image is 2196 $*$ 1740 pixels. There are some common pathological features of fundus images for glaucoma diagnosis, such as increased cup–disc ratio, retinal nerve fiber layer defect (RNFLD), peripapillary atrophy (PPA). In the retinal image, the optic disc is a vertical shallow ellipse, and the center of the optic disc is a white cup area, as shown in Fig. 9. The measurement of cup-to-disc ratio is the ratio of the area diameter of the optical cup-disc to the diameter of the optic disc [40]. Patients with glaucoma usually have a large cup-to-disc ratio; for example, when the ratio is greater than 0.5, glaucoma probably occur [4]. RNFLD is the lesion area in the fundus images (a roughly wedge-shaped region starting from the optic disc), which is one of the features to identify glaucoma [41]. Besides, PPA, a green area around the optic disc, is another major feature of glaucoma images [5]. We can find that these special features clearly appear in Fig. 9 (where Fig. 9b is a glaucoma image, while (a) is a normal condition).

**Fig. 10:**
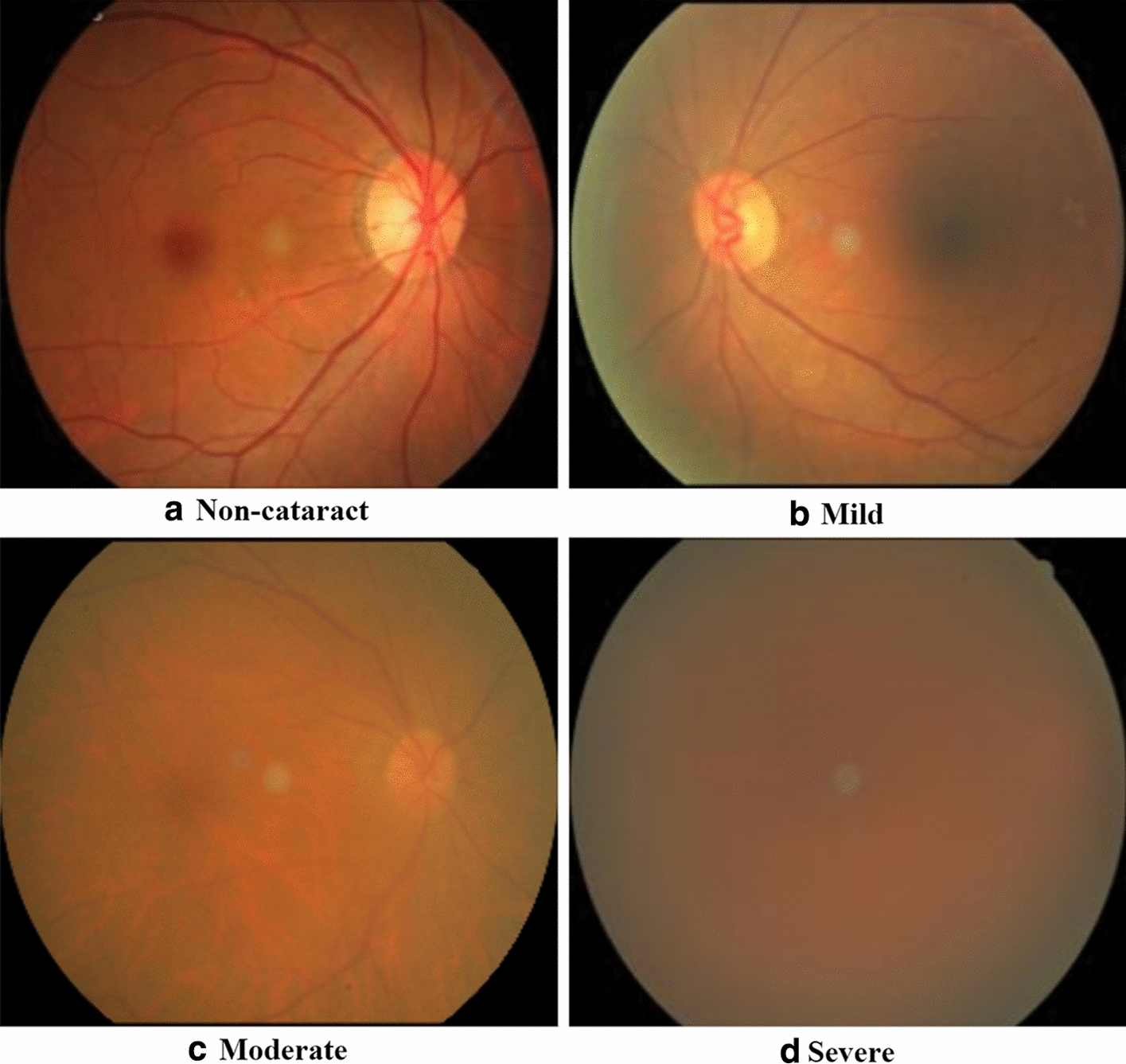
Fundus images of non-cataract and three different levels of cataracts

The cataract dataset used in our experiment comprises of 10463 retinal fundus images (3023 $*$ 2431 pixels), including non-cataract (3314), mild (2331), moderate (2245), and severe (2573) cataract images. Note that all diagnosis results are based on the unified grading standard [42–44]. Figure 10 shows four samples of cataract patients of varying degrees. Figure 10a is a cataract-free image, where the optic disc, large and small blood vessels are visible. Figure 10 (b) has fewer vascular details in moderate-to-mild cataract images, while in Fig. 10c, only large vessels and optic discs can be seen in moderate cataract images. In addition, in Fig. 10d, the severe cataract image, there is hardly anything to see. Based on these retinal fundus images, we can conclude that blood vessels and optic discs are the main references for cataract detection and classification.

### Transferring general features from similar ophthalmic dataset

As a kind of deep learning network, CNN is used in the field of image recognition to learn features automatically. Having a weight sharing network structure that is more similar to the biological neural network, CNN reduces the complexity of the network model. This advantage is more obvious when input of the network is multidimensional image. The kind of image can be used as the input of the network directly, thus avoiding the complex feature extraction and data reconstruction process of the traditional recognition algorithm. Therefore, we adopt an extension of a classic CNN network in [45], as the base model for transfer learning in our experiment. The base CNN network possesses a structure of seven layers: five convolutional layers and two fully connected (FC) layers. In the convolution layer, feature maps computed in the previous layer are convolved with a set of weights, the so-called filters. The generated feature maps are then passed through a nonlinearity unit, the rectified linear unit (RELU). Next, in the pooling layer, each feature map is subsampled with pooling over a contiguous region to produce the pooled maps. After performing convolution and pooling in the fifth layer, the output is then fed into fully connected layers to perform the classification. Besides, data augmentation and dropout methods are adopted to reduce overfitting.

**Fig. 11:**
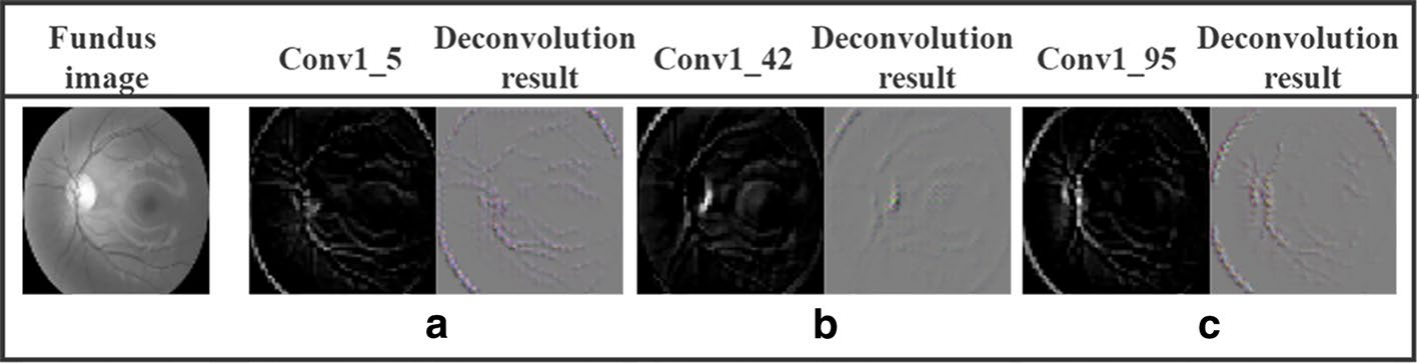
Visualization of feature maps and the corresponding deconvolution results in conv1 layer

In a trained CNN, features of the shallow layer are general, while those of the higher layer are task-specific; meanwhile, the middle layers transit gradually from general to specific, forming a hierarchical multilayer architecture [33]. The general layers are typically used to extract local edge features similar to Gabor filters. As shown in Fig. 11, we visualize feature maps and the corresponding deconvolution results of the first convolution layer. We can find that general features, such as edges and line segments of the fundus image, are extracted in different directions. Figure 11a–c tends to extract the edge contour features in − 45, 45, and 90 degree directions, respectively. When a pre-trained CNN structure is fine-tuned, the layers have to be frozen consecutively, so that any updated weight in the unfrozen shallower layers can be propagated to deeper layers. However, when transferring features from a less related source dataset, it may inversely hurt the transferability of general features.

Hence, rather than extracting general features from non-medical dataset, we transfer the weights of shallow layers, which are optimized to recognize the generalized structures in cataract dataset (shown in blue blocks in Fig. 1), and then retrain the weights of the deep layers with glaucoma dataset propagation. This strategy helps to identify the distinguishing features of glaucoma fundus images more accurately under limited supervision.

### Extracting specific features based on transfer induced attention

Specialization of deep layer neurons for the target task is based on general features. However, there still exists redundant regions in the fundus image when capturing specific features from general features of similar ophthalmic datasets. For example, the edge regions of the eyeball or other unrelated pathological areas are redundant for the glaucoma detection. To effectively refine specific features and remove irrelevant redundancy, we use a soft attention design across channels to replace the original CNN architecture.

As it is known, attention mechanism has been successfully applied in deep learning architecture, since it can locate the most salient parts of the features [46–49]. This meritorious property conforms to human visual perception: instead of trying to deal with the whole scene at the same time, human beings use a series of local glimpses to selectively focus on the prominent parts to better capture the visual structure [50]. As shown in the green block of Fig. 1, a transfer induced attention module is produced by utilizing the inter-channel relationship of general transferred features. In our transfer processing, each learned filter operates with a local receiving field; therefore, each unit of the transferred general features ${\mathbf {G}}$ is unable to exploit contextual information outside of this region. To tackle this issue, we use global average pooling (GAP) to compress the global spatial information, which helps to accelerate specific features extraction on glaucoma critical areas. Specifically, the element of ${\mathbf {o}}$ is generated by shrinking ${\mathbf {g}}$ through spatial dimensions $W \times H$:

3 $$o = {\text{GAP}}\left( \mathbf{g} \right) = \frac{1}{{W \times H}}\sum\limits_{{i = 1}}^{W} {\sum\limits_{{j = 1}}^{H} \mathbf{g} } (i,j).$$

GAP descriptor is then forwarded to FC layers which aims to recalibrate channel information adaptively:

4 $$\left. {{\mathbf{m}} = FC({\mathbf{o}}) = \sigma \left( {{\mathbf{W}}_{1} \left( {{\mathbf{W}}_{0} {\mathbf{o}}} \right)} \right)} \right),$$

where $\sigma$ refers to the sigmoid activation function, ${\mathbf {W}}_{1}$ and ${\mathbf {W}}_{0}$ are the FC layer weights, and ${\mathbf {m}}$ is our channel-wise attention map.

To get final specific feature ${\mathbf {P}}$, we reweight the original transferred general feature ${\mathbf {G}}$ with the channel attention map ${\mathbf {m}}$:

5 $$\begin{aligned} {\mathbf {P}}={\mathbf {G}} \otimes {\mathbf {m}}, \end{aligned}$$

where $\otimes$ denotes element-wise multiplication. During multiplication, the attention values are broadcasted accordingly. Besides, the attention-based specific feature ${\mathbf {P}}$ can help us highlight the discriminative regions by masking the original fundus image, which contributes to improve interpretability of our proposed model. When pre-training base CNN model on the source dataset, the cross-entropy $L_{ce}$ between the predicted label and its corresponding true label is defined as the loss function. When transferring general features to learn specific features, a new loss function is redefined by integrating three parts:

6 $${\text{Loss}} = L_{{{\text{ce}}}} \left( {{\mathbf{X}}_{s} ,{\mathbf{Y}}_{s} } \right) + L_{{ce}} \left( {{\mathbf{X}}_{t} ,{\mathbf{Y}}_{t} } \right) + \lambda L_{{{\text{Disc}}}} ,$$

where ${\mathbf {X}}_{s}$ and ${\mathbf {X}}_{t}$ refer to the sets of training images from the source and target datasets, respectively, and is $\lambda$ is non-negative regularization parameter. And the first and second parts represent the classification loss of corresponding dataset. The third term, discrepancy loss, aims to measure the distance of the feature vectors computed from the source and target datasets. Following the popular trend in transfer learning [51, 52], we rely on on the Maximum Mean Discrepancy (MMD) [53] to encode this distance. Supposed that $N_{s}$ and $N_{t}$ are the number of source and target samples respectively, then the $L_{Disc}$ is calculated through Eq. (5):

7 $$\begin{aligned} {\text {MMD}}^{2}\left( {\mathbf {m}}_{s}, {\mathbf {m}}_{t}\right) =\left\| \sum _{i=1}^{N_{s}} \frac{\phi \left( {\mathbf {m}}_{s}\right) }{N_{s}}-\sum _{j=1}^{N_{t}} \frac{\phi \left( {\mathbf {m}}_{t}\right) }{N_{t}}\right\| ^{2}, \end{aligned}$$

where $\phi (\cdot )$ denotes the mapping to RKHS. For network optimization, the mini-batch stochastic gradient descent (SGD) and back-propagation algorithm are used in this paper.

## Data Availability

The datasets generated and/or analyzed during the current study are not publicly available due to the clinical policy, but are available from the corresponding author on reasonable request.

## Abbreviations

TIA-Net: Transfer Induced Attention Network
HOS: higher order spectra
SVM: Support vector machine
CNN: Convolutional neural network
RNFLD: Retinal nerve fiber layer defect
PPA: Peripapillary atrophy
FC: Fully connected
ReLU: Rectified linear unit
GAP: Global average pooling
MMD: Maximum mean discrepancy
SGD: Stochastic gradient descent
AUC: Area under the curve
ROC: Receiver-operating characteristic
GPU: Graphics processing units
